# Cardiovascular magnetic resonance with an MR compatible pacemaker

**DOI:** 10.1186/1532-429X-15-18

**Published:** 2013-02-15

**Authors:** Anita R Bhandiwad, Kristopher W Cummings, Michael Crowley, Pamela K Woodard

**Affiliations:** 1 Division of Cardiology, Department of Medicine, Washington University School of Medicine, 1020 N. Mason Road, Suite 100, Saint Louis, MO, 63141, USA; 2 Mallinckrodt Institute of Radiology, Washington University School of Medicine, Saint Louis, MO, USA

**Keywords:** MRI-conditional pacemaker, Cardiovascular magnetic resonance, Revo pacer

## Abstract

Magnetic resonance imaging (MRI) within FDA guidelines for the MRI-conditional pacemaker precludes placing the heart at the center of the magnet’s bore. This in effect appears to preclude cardiovascular MR. In this manuscript, we describe a protocol for cardiovascular MR of patients with a Revo pacemaker system while operating within FDA guidelines, and the first US case of cardiovascular MR in a patient with a Revo MRI-conditional pacing system despite position constraints.

## Background

Several million people are estimated to currently have cardiac implantable electronic devices (CIEDs), including pacemakers and implanted cardioverter defibrillators (ICD). This number may continue to grow with expanded indications for heart failure and primary prevention. Historically, CIEDs have been considered to be absolute contraindications for magnetic resonance imaging (MRI). However, there is an estimated 50–75% probability that MRI will be indicated for a patient over the device lifetime [1].

There are several concerns regarding the interaction of these devices with the static and pulsed magnetic field. First, the generator or leads may be displaced, although translational forces and torque on the leads have been shown to be small at 1.5 Tesla field strength [2]. Next, the behavior of the device and programming may be altered. For instance, pacemakers may change to asynchronous pacing mode or an ICD may temporarily suspend detection and therapies of ventricular tachyarrhythmias. Pulsed radiofrequency from the MRI may induce voltage pulses that result in oversensing of electrical signals in the magnetic field. Alternatively, pacing inhibition in a pacemaker- dependent patient, unintended cardiac stimulation, and possible inappropriate shocks could result [1,3,4]. Finally, radiofrequency-induced heating of myocardial tissue near the pacemaker lead tip has the potential to cause thermal injury with resultant pacing threshold deterioration or even atrial or ventricular perforation [2]. Therefore, even nonfunctioning leads left without connection to a generator or epicardial leads have been considered a contraindication to MRI.

Device programming can mitigate some of these potential risks. However, electrical reset can occur in up to 6% of pacemaker patients undergoing MRI upon exposure to a magnetic field that overrides programming changes [2,5]. Pacemaker inhibition could lead to bradycardia/asystole, or competitive rhythms may occur that can induce fatal tachyarrhythmias [2,4].

Although MR conditional pacemaker, ICD, cardiac resynchronization therapy pacemaker and defibrillator systems are available in Europe and some in Asia, most are investigational in the United States (Biotronik, Berlin, Germany, and St. Jude Medical, St. Paul, Minnesota). Most recently, development and Food and Drug Administration (FDA) approval of the MRI-conditional device, the Medtronic Revo pacing system (Medtronic, Minneapolis, Minnesota), has been a notable advance. Conditions for scanning patients with the Revo system within FDA guidelines include a 6-week delay after pacemaker implantation, 1.5 T static magnetic field strength, maximum specific absorption rate (SAR) of 2 W/kg for each sequence, and maximum slew rate of 200 T/m/s. MR scanning within FDA guidelines for the device precludes placing the heart at the magnet isocenter (center of bore). However, the pacemaker system may pass through isocenter during table positioning. Isocenter must be above the superior surface of the C1 vertebra or below the inferior surface of the vertebral body of T12 [4]. This in effect appears to preclude cardiovascular MR (CMR). In this manuscript, we describe a protocol for CMR of patients with a Revo pacemaker system while operating within FDA guidelines, and the first US case of CMR in a patient with a Revo MRI-conditional pacing system despite position constraints.

## Case presentation

A 34-year-old woman had a history of an anterior myocardial infarction following spontaneous left anterior descending coronary artery dissection 11 days postpar-tum at age 28 and subsequently underwent cardiac transplantation. She was found to have chronic inflammation and episodes of acute rejection on endomyocardial biopsies. Echocardiography demonstrated abnormal diastolic function with restrictive filling and course was notable for development of clinical heart failure requiring hospitalization. Biopsies confirmed post-transplant lymphoproliferative disorder (PTLD) consistent with plasmacytoma. CMR demonstrated patchy areas of late gadolinium enhancement (LGE) in the inferior, inferoseptal and lateral walls of the left ventricle consistent with infiltrative process. The patient’s course was further complicated by development of sinus node dysfunction with sinus pauses of 4.5 seconds. Pacemaker implantation was advised. MRI-compatible dual-chamber system was implanted due to the anticipated need for future CMR studies to follow cardiac allograft involvement with chemotherapy [6].

**Figure 1:**
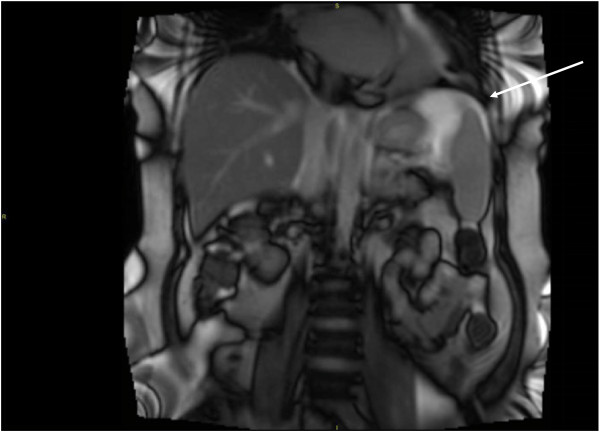
**SSFP coronal scout with isocenter below T12.** Position confirmed by inferior rib while placing patient in scanner. Note the relatively higher signal at the image isocenter (arrow).

The patient was referred for CMR 6 months after pacer implantation. The scan was performed on 1.5 T whole body scanner (TIM Symphony, Siemens Medical Systems, Malvern, NJ), slew rate 125 T/m/s. A cardiologist, radiologist and MRI physicist were present. Isocenter was placed inferior to T12, determined by the inferior rib (Figure 1). Minor modifications to the flip angle of the cine steady-state free precession (SSFP) sequences were made to maintain SAR < 2 W/kg. The table position was set to “FIXED” to prevent default movement. Prior to scanning, the device was interrogated: atrial lead impedance 568 ohms, ventricular lead impedance 472 ohms, atrial lead capture threshold 1 V at 0.4 ms, ventricular lead capture threshold 1 V at 0.4 ms, P-wave amplitude sensing 3.4 mV, R-wave amplitude sensing 10.3 mV. The device was switched to “SureScan On” and ODO mode. Following completion of imaging, device was set to “SureScan Off” and DDD mode, and interrogation showed no significant change in parameters: atrial lead impedance 544 ohms, ventricular lead impedance 472 ohms, atrial lead capture threshold 1 V at 0.4 ms, ventricular lead capture threshold 1 V at 0.4 ms, P-wave amplitude sensing 3.5 mV, R-wave amplitude sensing 9.9 mV. The patient was monitored throughout the study by telemetry, blood pressure, and voice communication. The patient had no complaints during scanning.

**Figure 2:**
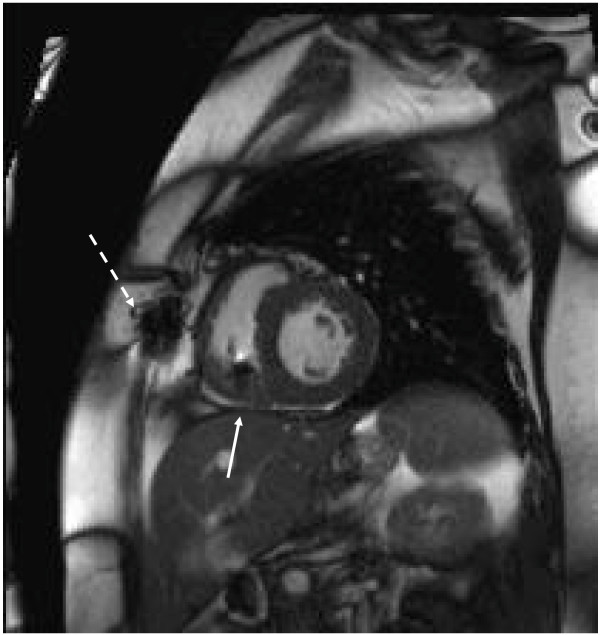
**Still-frame from cine steady-state free precession image.** Localized artifact from sternal wires (dashed arrow) and pacemaker lead in right ventricle (solid arrow).

**Figure 3:**
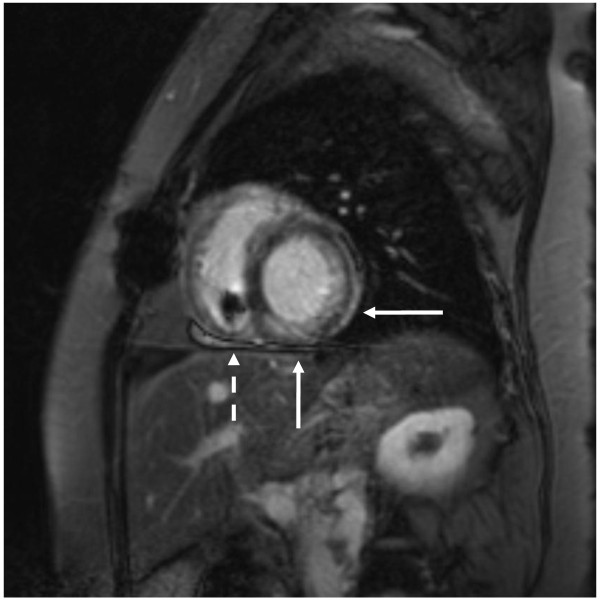
**Late gadolinium enhancement images demonstrating enhancement in the inferior and lateral walls.** Findings consistent with infiltrative process and biopsy-confirmed PTLD (solid arrows), and pacemaker lead in right ventricle (dashed arrow).

A complete CMR study without and with contrast was performed with black blood half-fourier acquisition single-shot turbo spin-echo (HASTE) scout imaging, cine SSFP sequences (TR 3.0 ms, TE 1.3 ms, flip angle < 90° modified to maintain SAR <2 W/Kg) in the 2 and 4-chamber long-axis and short axis cardiac planes, followed by segmented gradient-recalled phase sensitive inversion recovery (PSIR) LGE short and long axis imaging (TR 46 ms, TE 3.4 msec, flip angle 15°, IR time 280 msec) performed after intravenous administration of 0.2 mmol/Kg gadoversetamide (Optimark, Covidien, St. Louis, MO). These images demonstrated patchy LGE of the left ventricular inferior and lateral walls consistent with infiltrative process and known PTLD. Localized artifact from the pacemaker lead in the right ventricle was present without effect on interpretability of images (Figures 2 and 3).

**Figure 4:**
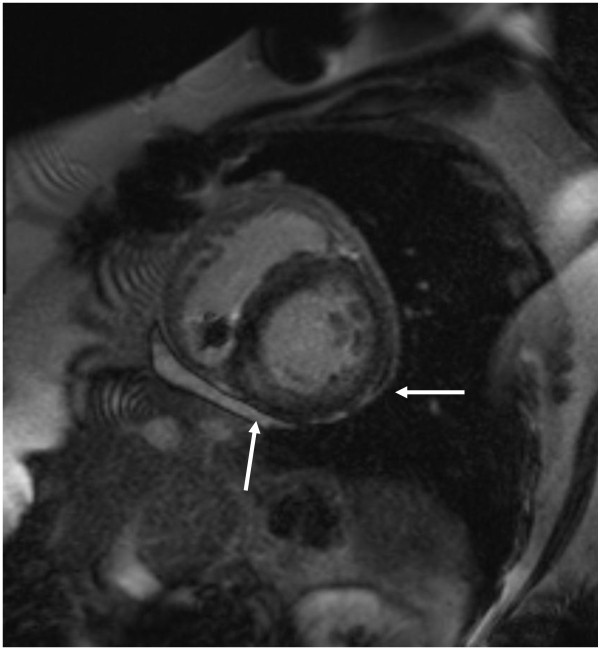
**Late gadolinium enhancement image on CMR study 4 months later.** Slight improvement in inferior and lateral enhancement (solid arrows).

Four months later the patient was referred again for CMR to assess response to therapy. A similar protocol as described above was performed. These images demonstrated slight interval decrease in enhancement pattern (Figure 4).

## Conclusion

Quarta et al. described CMR with the MRI-conditional pacing system performed in Europe where positioning restrictions are not required [7]. Following appropriate U.S. imaging protocols for this device, diagnostic quality cardiac images can be obtained despite position of isocenter remote from the heart. Localized artifact from leads does not compromise image interpretability. The MRI-conditional pacemaker system may allow the benefits of MRI to be more accessible to pacemaker patients.

## Consent

Written informed consent was obtained from the patient for publication of this case report and any accompanying images. A copy of the written consent is available for review by the Editor-in-Chief of this journal.

## Abbreviations

(CIEDs): Cardiac implantable electronic devices; (ICD): Implanted cardioverter defibrillators; (MRI): Magnetic resonance imaging; (CMR): Cardiovascular magnetic resonance; (FDA): Food and Drug Administration; (SAR): Specific absorption rate; (PTLD): Post-transplant lymphoproliferative disorder; (LGE): Late gadolinium enhancement; (SSFP): Steady-state free precession; (PSIR): Phase sensitive inversion recovery.

## Competing interests

PKW- Medtronic, consultant. The other authors have no disclosures or conflicts of interest.

## Authors’ contributions

ARB provided cardiology supervision during scanning, reviewed device interrogations, assisted in image acquisition and interpretation, and drafted the manuscript. KWC assisted with planning scan protocol. MC, a physicist, modified sequences for SAR limitations. PKW planned scan protocol, assisted in image acquisition and interpretation, and helped to draft the manuscript. All authors read and approved the final manuscript.
